# Molecular detection of tick-borne rickettsial and protozoan pathogens in domestic dogs from Turkey

**DOI:** 10.1186/s13071-015-0763-z

**Published:** 2015-03-14

**Authors:** Munir Aktas, Sezayi Özübek, Kürşat Altay, Neval Duygu Sayin Ipek, İbrahim Balkaya, Armagan Erdem Utuk, Akın Kırbas, Sami Şimsek, Nazir Dumanlı

**Affiliations:** Department of Parasitology, College of Veterinary Medicine, Firat University, 23119 Elazig, Turkey; Department of Parasitology, College of Veterinary Medicine, Cumhuriyet University, Sivas, Turkey; Department of Parasitology, College of Veterinary Medicine, Dicle University, Diyarbakır, Turkey; Department of Parasitology, College of Veterinary Medicine, Atatürk University, Erzurum, Turkey; Department of Parasitology, Ceyhan Veterinary Medicine, Cukurova University, Adana, Turkey; Department of Internal Medicine, College of Veterinary Medicine, Atatürk University, Erzurum, Turkey

**Keywords:** Dog, *Babesia canis canis*, *Anaplasma platys*, *Ehrlichia canis*, *Theileria annulata*, RLB

## Abstract

**Background:**

Canine tick-borne parasites have emerged in recent years, showing a wider geographic distribution and increased global prevalence. In addition to their veterinary importance, domestic dogs play an important role in the transmission cycles of some agents by acting as reservoirs and sentinels. This study investigated *Babesia*, *Theileria*, *Anaplasma*, and *Ehrlichia* species in asymptomatic dogs in ten provinces of Turkey.

**Methods:**

DNA obtained from blood samples collected from 757 domestic dogs (243 stray, 351 shelter, 163 pet) of both sexes and various ages were evaluated using PCR and reverse line blotting (RLB) assays.

**Results:**

Of the 757 dogs tested, 41 (5.4%) were found to be infected with one or more parasites. *Ehrlichia canis* (37/757, 4.9%) was the most common canine tick-borne pathogen, followed by *Anaplasma platys* (4/757, 0.5%). *Babesia canis* and *Theileria annulata* were each detected in 1 (0.13%) sample. Combined infection of *E. canis* and *A. platys* was detected in 2 (0.3%) samples. The prevalence of tick-borne pathogens was higher in adult dogs (6.8%) than in those under one year old (3.1%). Difference in infection rate of male and female dogs was not significant. Pet dogs had a lower prevalence of infection (1.2%) compared to stray (7.4%) and shelter dogs (6%) although the difference between stray and shelter dogs was not significant.

**Conclusions:**

*Babesia canis*, *T. annulata*, *A. platys*, and *E. canis* species were identified at the molecular level in dogs in several provinces of Turkey, with *E. canis* being the most common species among tick-borne pathogens. Detailed studies should be conducted regarding the existence and prevalence of *B. canis* and *Dermacentor reticulatus* in eastern Turkey.

## Background

Rickettsia and protozoa are transmitted by ixodid ticks, and cause both clinical and subclinical infections of their hosts. Among these, babesiosis, anaplasmosis, and ehrlichiosis are significant infectious diseases of dogs [1]. Canine babesiosis is characterized by fever, anemia, hemoglobinuria, thrombocytopenia, jaundice, and functional disorders of organs [2]. Several species of *Babesia* may infect dogs, including large (e.g., *B. canis*, *B. rossi*, *B. vogeli*) and small (e.g., *B. gibsoni*, *B. conradae*) forms of *Babesia* [3]. The geographic distribution of these species is closely related to the distribution of vector ticks. *Babesia canis* occurs in countries with a temperate climate [4], *B. rossi* is found in South Africa [5], and *B. vogeli* in regions in which *Rhipicephalus sanguineus* sensu lato exists [3]. These species are transmitted by *Dermacentor, Rhipicephalus*, and *Haemaphysalis* ticks [6].

Anaplasmosis, caused by *Anaplasma phagocytophilum* and *Anaplasma platys*, and ehrlichiosis, caused by *Ehrlichia canis*, *Ehrlichia chaffeensis*, *Ehrlichia ewingii*, and *Ehrlichia muris*, are emerging infectious diseases affecting dogs in many parts of the world and can be manifested as acute or non-clinical infections [7]. Weight loss, anorexia, pale mucous membranes, high fever, lethargy, lymphadenopathy, and splenomegaly are the most commonly observed clinical signs [8]. *Ehrlichia canis* is transmitted by *Rh. sanguineus* s.l. and has also been experimentally transmitted by some *Dermacentor* species [9]. The DNA of *A. platys* was reported in *Rh. sanguineus* s.l. ticks [5].

Tick-borne diseases can be diagnosed from clinical signs and microscopic examination of stained blood smears. However, as the morphology of the pathogens in the host cell is similar, species cannot be distinguished by microscopic examination [10]. Microscopic examination of stained blood smears has very limited sensitivity [11-13]. In addition, in-clinic serology tests do not differentiate active infection from prior exposure [14], therefore, molecular techniques have become the preferred method for detection of tick-borne hemoparasites in vertebrates and ticks [15-21].

Canine tick-borne disease caused by *Babesia*, *Anaplasma*, and *Ehrlichia* have been reported in Turkey [22-26], but there is limited information regarding prevalence of these pathogens. The objective of this survey was to investigate the frequency and distribution of tick-borne protozoan and bacterial pathogens in asymptomatic domestic dogs from ten provinces of Turkey.

## Methods

### Study site and collection of samples

**Figure 1:**
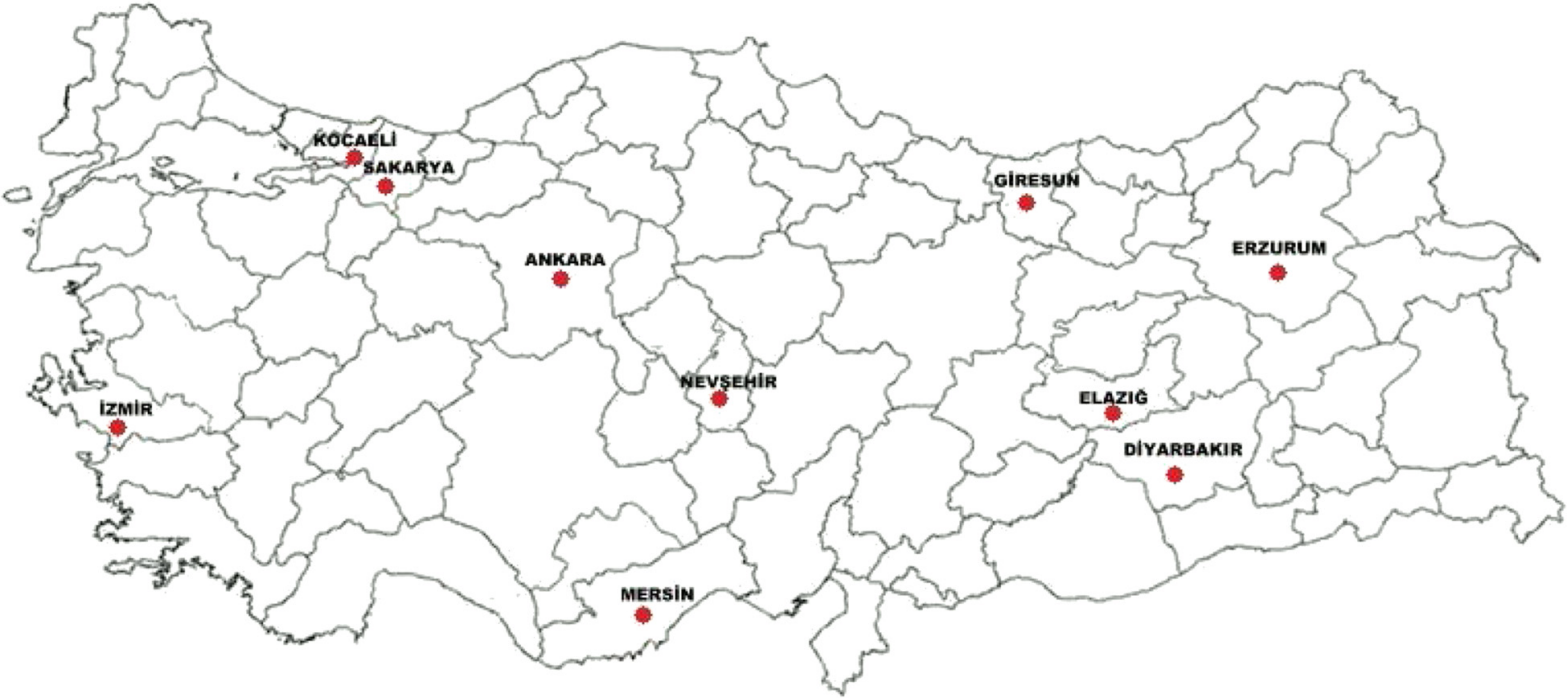
**Province boundaries of Turkey and survey locations.**

This study was conducted on asymptomatic domestic dogs in coastal (Sakarya, Kocaeli, Mersin, Giresun, and İzmir) and inland (Elazig, Diyarbakır, Erzurum, Ankara, Nevşehir) provinces of Turkey (Figure 1). The coastal provinces have temperate oceanic climate (with warm, wet summers and cool to cold, wet winters) and Mediterranean climate (with hot, dry summers and mild to cool, wet winters). At lower levels of these provinces, the forest is mainly deciduous, often associated with evergreen shrubs, but at higher levels conifers increase or become dominant. The inland provinces have a continental climate (with hot summers and colder winters), and steppic habitats represent the major natural vegetation. The average annual precipitation in Aegean (İzmir), Mediterranean (Mersin) and Black Sea (Sakarya, Kocaeli, Giresun) coasts varies from 580 to 2200 mm, whereas 400 mm in the inland provinces [27]. The altitude of the study sites ranges from 2 (İzmir) to 1890 (Erzurum) m above sea level. There is no information regarding the estimated dog population in both coastal and inland provinces. Hovewer, at least 49 dogs in each province were sampled.

Blood samples (3 mL) were collected in EDTA tubes from 757 domestic dogs (243 stray, 351 from shelters, 163 pets). The study was conducted in cooperation with municipal dog shelters, Fırat University Veterinary Faculty, and private veterinary clinics. Sample collection was conducted from June 2010 to October 2012. Blood samples were collected from both male and female dogs of different breeds and various ages. Gender, age, and origin (stray, shelter, pet) of the dogs were recorded at sampling time. Age was estimated from body size and teeth, with those aged 6 months to 1 year designated as young, and those from 1 to 7 years considered adults. Based on behavior, all dogs appeared healthy, but detailed clinical examinations were not conducted.

### Ethical approval

This study was approved by Firat University experimental animal ethic committee (approved protocol no. 16.02.2010-15).

### Microscopic examination

Immediately after sample collection, 348 thin blood smears were prepared for microscopic examination. The smears were fixed with methanol for 5 min and then stained with 5% May-Grunwald Giemsa in buffer solution for 30 min. The stained slides were examined under oil immersion at 1000× on a Nikon microscope for the presence of piroplasms (*Babesia*, *Theileria*) and inclusion bodies (*Anaplasma*, *Ehrlichia*). This was done by a person who was blinded to the molecular results.

### DNA isolation and PCR amplification

Genomic DNA extraction was performed using QIAamp DNA Blood Mini kits (Qiagen, Hilden, Germany) according to the manufacturer’s protocol. Negative control purifications using sterilized de-ionized water were performed to monitor cross-contamination for each batch of 18 samples. A total of 15 DNA samples were randomly selected for the quantification. DNA concentrations (ng/μL) and purity (*A*_260__nm_/ *A*_280 nm_) were determined by spectrophotometry (NanoDrop® ND- 2000 UV/Vis Spectrophotometer, Thermo Fisher Scientific Inc., Wilmington, Delaware, USA). In order to minimize potential risks of contamination, DNA extractions, PCR preparation, PCR amplification, and agarose gel electrophoresis were performed in separate rooms. Genus-specific primers, RLBF2/RLBR2, were used to amplify a fragment of 460–540 bp of the 18S SSU rRNA gene of the V4 region of *Theileria* and *Babesia* species [28]. For identification of *Anaplasma* and *Ehrlichia* species, the primers 16S8FE and BGA1B were used to amplify a fragment of approximately 500 bp of the 16S rRNA gene of the V1 region of *Anaplasma* and *Ehrlichia* spp., as described by Schouls et al. [15]. The PCR reactions were performed in PCR Sprint (Thermo Electron Corporation, USA), using a touchdown PCR program as previously described [29].

For positive control of *B. canis*, PCR positive amplicon obtained a dog naturally infected with *Babesia* spp. were purified and sequenced to identify amplified organisms. Sequencing result indicated that the amplicon was similar to the corresponding *B. canis* sequences deposited in GenBank. This template was used as a positive control DNA for *B. canis*. DNA from *E. canis* which tested positive by RLB and DNA sequencing (KF034789) obtained in a previous study [26] were used as positve control. In order to determine the detection limit of the PCR based RLB assay, serial ten-fold dilutions (from 10^−1^ to 10^−10^) of *E. canis* and *B. canis canis* positive control DNA (starting at 22.7 and 11.8 ng/μl, respectively) were prepared in sterilized de-ionized water and tested. The lowest DNA concentration that yielded an RLB signal was considered as the limit of detection of the PCR based RLB assay. Positive controls with the lowest detection limit were included in every assay run. Sterilized de-ionized water was used as negative control.

### Reverse line blotting (RLB)

To detect *Theileria*, *Babesia*, *Anaplasma*, and *Ehrlichia* species, a reverse line blot (RLB) hybridization was performed on the PCR products, as described previously [18]. Briefly, to 20 μL of the PCR products, 2X SSPE/0.1% SDS was added to a final volume of 150 μL and held in the Thermal Cycler at 99°C for 10 min and denatured for RLB hybridization. Catch-all and species specific probes were attached to the membrane in order to identify common tick-borne *Babesia*, *Theileria*, *Anaplasma* and *Ehrlichia*. Probes were provided by the Midland Certified Reagent Company (Texas, USA).

### Sequencing

In order to confirm the results obtained by PCR and RLB, a total of 10 amplicons consist of *B. canis* (n = 1), *T. annulata* (n = 1), *A. platys* (n = 2) and *E. canis* (n = 6) were chosen for sequencing. They were selected on the basin of geographic locations where samples were collected (Mersin, Giresun, İzmir, Diyarbakır, Erzurum). The generated DNA fragments were purified with a PCR purification kit (Qiagen, Hilden, Germany) and sequenced directly in an automated DNA sequencer. DNA sequences obtained were evaluated with Chromas Lite software, version 2.01 (Technelysium Pty Ltd) and compared for similarity to sequences deposited in GenBank. Primer was deleted from DNA sequences prior to BLAST comparison.

### Statistical analysis

Fisher’s exact test was used to evaluate differences among prevalence of tick-borne pathogens and host gender, age, and origin. The test was performed using Epi Info software program, version 6. Statistical significance was defined as p < 0.05.

## Results

### DNA quantification and sensitivity of the PCR based RLB

The DNA concentration ranged from 11.4 to 32.7 (median, 19.9) ng/μL. The A260 nm/A280 nm ratios of the same extracts ranged from 1.30 to 2.12 (median, 1.60). In the positive control DNA serial dilution test, the detection limit of the assay was found to be 10^−3^ and 10^−6^ for *E. canis* and *B. canis*, respectively.

### Prevalence of tick-borne pathogens in dogs

With blood smear examination, all slides were negative for piroplasms (*Babesia*/*Theileria*) and inclusion bodies (*Anaplasma*/*Ehrlichia*). *Babesia*/*Theileria* spp. DNA (hypervariable V4 region of the 18S rRNA gene) was amplified by RLBF2/RLBR2 primers in 2/757 (0.3%) dogs, while *Anaplasma/Ehrlichia* spp. DNA (V1 region of the 16S rRNA gene) was amplified by 16S8FE/B-GA1B primers in 39/757 (5.1%) dogs. PCR performed on a negative control did not yield any product on agarose gel. All PCR positive samples showed positive signals with the corresponding specific probes linked on RLB membrane.

Frequency of tick-borne pathogen detected by RLB from samples at locations is presented in Table 1. While the highest number of positive samples was obtained from the province of Giresun with 28.0%, the lowest was Erzurum in 0.8%. No pathogen was detected in the province of Elazig, Ankara, Nevşehir, Adapazarı and Izmit.

**Table 1:** **The frequency of tick-borne pathogen detected by RLB from samples at locations**

**Province**	**No.tested**	**Identified pathogen**				**Total**
		***B. canis***	***T. annulata***	***A.platys***	***E. canis***	
Elazığ	150	-	-	-	-	-
Diyarbakır	63	-	-	-	10	10 (15.9%)
Erzurum	126	1	-	-	-	1 (0.8%)
Ankara	49	-	-	-	-	-
Nevşehir	51	-	-	-	-	-
Adapazarı	65	-	-	-	-	-
İzmit	69	-		-	-	-
Mersin	74	-	1	2	5	6 (8.1%)*
Giresun	50	-	-	-	14	14 (28.0%)
İzmir	60	-	-	2	8	10 (16.7%)
Total	757	1 (0.1%)	1 (0.1%)	4 (0.5%)	37 (4.9%)	41 (5.4%)

* Two samples were co-infected with *A.platys* and *E. canis.*

Prevalence of single and combined tick-borne infections is shown in Table 2. Using RLB, 41/757 (5.4%) of the dogs were found to be infected with one or more parasites of four tick-borne pathogens: *Babesia canis*, *Theileria annulata*, *A. platys*, and *E. canis. Ehrlichia canis* (4.9%) was the most frequent, followed by *A. platys* (0.5%). *Babesia canis* and *T. annulata* were each detected in 1 (0.1%) dog. Mixed infections were also found; the co-existence of *E. canis* and *A. platys* was detected in 2 (0.3%) dogs.

**Table 2:** **Distribution and frequency a of tick-borne pathogens in domestic dogs, detected by DNA amplification and reverse line blotting**

**Infection status**	**Identified pathogens**	**n**	**%**
Single infection	*Ehrlichia canis*	35	4.6
	*Anaplasma platys*	2	0.3
	*Babesia canis canis*	1	0.1
	*Theileria annulata*	1	0.1
Mixed infection	*Ehrlichia canis* + *Anaplasma platys*	2	0.3
PCR-RLB positive	any parasite (*Theileria/Babesia, Anaplasma*/*Ehrlichia*)	41	5.4
Negative		716	94.6
Total		757	100

The distribution of tick-borne infections according to the sex, age, and location of the animal (stray, shelter, pet) is given in Table 3. No significant differences were observed between male and female dogs (*P =* 0.976). The frequency of infection was higher in adult dogs (*P =* 0.040). The frequency rate was 7.4% (25/454), 6% (31/351) and 1.2% (2/163) in stray, shelter, and pet dogs, respectively. These results show lower infection prevalence in pets compared to the stray (*P =* 0.027) and shelter dogs (*P =* 0.009). The difference in infection rate of stray and shelter dogs was not significant (*P =* 0.602).

**Table 3:** **Comparison of tick-borne pathogen ferquency obtained in dogs according to the gender, age and origin (stray, shelter, pet)**

	**gender**		**age**		**origin (location of the animal)**		
	**female**	**male**	**young**	**adult**	**stray**	**shelter**	**pet**
No. of samples	454	303	290	467	243	351	163
Positive	25 (5.5%)	16 (5.3%)	9 (3.1%)	32 (6.8%)	18 (%7,4)	21 (6%)	2 (1.2%)
p(F)*	p(F) = 0.976 (NS)		p(F) = 0.040		p(F) = 0.602 (NS)		p(F) = 0.027
							p(F) = 0.009

* Fisher’s exact test; p value; NS, Not significant.

A total of 10 samples which were positive for corresponding species-specific probes were sequenced in this study. All the sequences revealed an agent species consistent with the RLB result. Of these, five partial sequences have been deposited in GenBank under the following accession numbers: KF038322, KF038320, KP745630-KP745632.

## Discussion

Tick-borne protozoa and bacteria cause clinical infections in dogs in many regions of the world, depending on distribution of the vector tick. *Ehrlichia canis* (4.9%) was the most common canine tick-borne pathogen found in this study. The finding is in agreement with a previous report that *Rh. sanguineus* s.l. is the most prevalent tick species in infested dogs [30]. In the Aegean region of Turkey, *E. canis*, *A. platys*, and *A. phagocytophilium* were observed in 41.5%, 39.4%, and 52% of sampled dogs, respectively [22]. The low prevalence values were reported here for rickettsial infections (4.9% for *E. canis* and 0.5% for *A. platys*) when compared to the findings of the previous study [22]. Furthermore, the RLB assay conducted here contained a probe to detect *A. phagocytophilum*, but the results were negative. There was no information regarding the clinical status of the dogs sampled in the Aegean study [22], but healthy dogs were sampled in the present study and the sample type might therefore be responsible for the great differences between both studies. Another plausible explanation may be the lack of data about the analytical sensitivities of the methodologies used in both studies. Also, seasonal and geographical differences might have contributed to this.

Tick transmitted infections generally involve multiple pathogens [31,32]. In a study conducted in the Aegean region, 8 of 10 dogs that were naturally infected with *Hepatozoon canis* were found to also exhibit coexisting *E. canis*, *A. platys*, and *A. phagocytophilum* infections [33]. In this study, mixed infections of *A. platys* and *E. canis* were observed.

Canine babesiosis caused by *B. canis* is commonly observed in central Europe and Russia. Its distribution has expanded towards the north in recent years, and it is reported in Norway and Holland [3]. *Babesia canis* is transmitted by *Dermacentor reticulatus*, and the distribution area of the parasite is directly related to the presence of this tick species [2]. Although *B. gibsoni* has been observed in dogs in Turkey [24], neither babesiosis associated with *B. canis* nor the existence of *D. reticulatus* was reported in Turkey until 2013, when both the parasite and its vector were reported in 3 dogs in the East Anatolian region of Turkey [25]. In the present study, *B. canis* was observed in one of the blood samples collected from this region. These findings demonstrate that *B. canis* and *D. reticulatus* have formed a permanent population, at least in the eastern part of Turkey.

Tropical theileriosis caused by *T. annulata* is a widespread disease of cattle in Turkey [34,35]. This protozoon is transmitted by *Hyalomma* ticks. In this study, *T. annulata* was detected in a single dog. The finding is not surprising, as previous works have reported some piroplasm species in mammals other than their specific hosts [36-40]. Moreover, the infection by *T. annulata* has been previously reported in dogs from Iran [41].

In this study, the frequency of tick-borne infections was higher in adult dogs (6.85%) than young (3.10%), similar to findings of de Miranda et al. [42]. This can be explained by the fact that adult animals have greater exposure to tick-borne pathogens than do the young animals, and that the pathogens persist in the host for an extended time after the acute infection is resolved. On the other hand, some studies have reported that the prevalence of infection is not associated with the age of the host [43], implying that factors such as vector intensity and geographic distribution also influence the prevalence of tick-borne infections.

## Conclusions

*Babesia canis*, *T. annulata*, *A. platys*, and *E. canis* species were identified at the molecular level in dogs in several provinces of Turkey, with *E. canis* being the most common species among tick-borne pathogens. Detailed studies should be conducted regarding the existence and prevalence of *B. canis* and *D. reticulatus* in eastern Turkey.
